# Knowledge and acceptability of the rectal treatment route in Laos and its application for pre-referral emergency malaria treatment

**DOI:** 10.1186/1475-2875-9-342

**Published:** 2010-11-27

**Authors:** Southisouk Inthavilay, Thierry Franchard, Yang Meimei, Elizabeth A Ashley, Hubert Barennes

**Affiliations:** 1 Institut Francophone pour la Médecine Tropicale, Vientiane, Laos; 2 Mahidol-Oxford Tropical Medicine Research Unit (MORU), Mahidol University, Bangkok, Thailand

## Abstract

**Background:**

Rectal artesunate has been shown to reduce death and disability from severe malaria caused by delays in reaching facilities capable of providing appropriate treatment.

Acceptability of this mode of drug delivery in Laos is not known. In 2009 the acceptability of rectal treatments was evaluated among the general Lao population and Lao doctors in a national survey.

**Methods:**

A cross sectional survey was performed of 985 households selected through a multi-stage random sampling process from 85 villages in 12/18 provinces and of 315 health staff randomly selected at each administrative level.

**Results:**

Out of 985 families, 9% had used the rectal route to treat children (the main indication was seizures or constipation). The population considered it less effective than other routes. Other concerns raised included pain (28%), discomfort for children (40%) and the possibility of other side effects (20%). Of 300 health staff surveyed (nurses 44%, doctors 66%), only 51% had already used the rectal route with a suppository, mostly to treat fever (76%). Health staff working in provincial hospitals had more experience of using the rectal route than those in urban areas. The majority (92%) were keen to use the rectal route to treat malaria although oral and intramuscular routes were preferred and considered to be more efficacious.

**Discussion and conclusion:**

Use of rectal treatments is uncommon in Laos and generally not considered to be very effective. This view is shared by the population and health care workers. More information and training are needed to convince the population and health staff of the efficacy and advantages of the rectal route for malaria treatment.

## Background

In order to reduce malaria-related mortality improved access to effective treatments is needed. In endemic countries, malaria occurs mainly in rural areas far from health centres and hospitals. The need for hospital referral of severe cases causes further treatment delays since patients are often too sick to tolerate oral medication and no alternatives are available. The possibility to administer anti-malarials via the rectal route has been studied for many years, first with quinine, and more recently with the artemisinin derivatives[1-3]. In 2009, Gomes and colleagues reported the benefits of pre-referral rectal artesunate deployed in a large trial in Bangladesh and Tanzania[4]. A single pre-referral artesunate suppository reduced the risk of death or permanent disability in patients reaching a clinic with facilities for injection, if the travel time was more than 6 to 15 hours. The investigators did not encounter any problems related to lack of acceptability of the treatment. Studies from Mali and Zambia have also shown good acceptability of the rectal route for malaria treatment [5,6].

In Laos, rectal artesunate was incorporated into the national treatment policy for malaria in 2004, however its use was abandoned as this mode of medicine administration was not found acceptable by primary health workers and patients [7].

The objective of this study was to investigate the acceptability of the rectal route among Lao people, and to explore any use of the rectal route for traditional remedies. The study was incorporated into a larger survey of nutrition and epilepsy. Members of the general population were surveyed as well as a random sample of health care workers.

### Study area and population

Laos is a multi-ethnic and multilingual country with more than 45 languages. The official literacy rate in Laos is 73% with a disparity between urban areas (89%) and rural areas (54%).

## Methods

### Population survey

**Figure 1:**
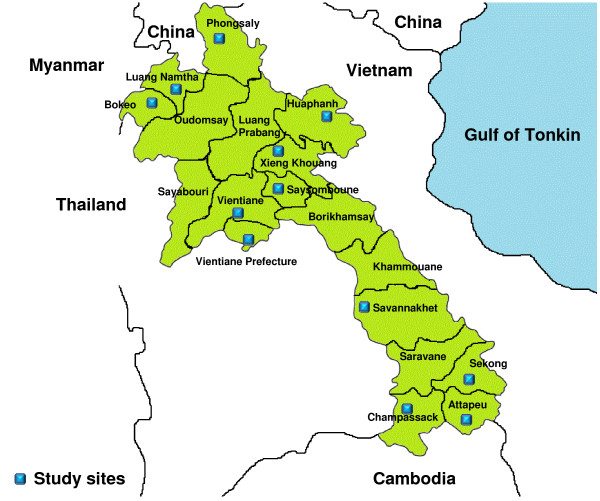
**Map of study site in 12/18 provinces of Laos**.

A randomised two-stage sampling survey of adults > 18 years old was conducted in 85 villages in 12/18 Lao provinces (Figure 1). Prior to the investigation, one district was randomly selected from each of the 12 provinces. A multi-stage random sampling approach was applied within each selected district. At the first stage, eight villages were selected from the list of all villages in each selected district and at the second stage, 11 households were selected from the list of households in every village. Random numbers were used to select the villages and households using the 2005 Census list. If the selected household was not available, the house next door was selected, if it complied with the inclusion criteria. One adult (> 18y) per household was chosen to be interviewed in the Lao language from all household members present. In addition, doctors and nurses in the medical wards of 12 provincial hospitals, 11 district hospitals and 31 health centres were interviewed.

### Data collection

A 32-question and a 23-question questionnaire were employed to ascertain the population and health staff's knowledge and experience of using the rectal route. The questionnaires were pre-tested in the general population in Vientiane. Questionnaires were administered to the general population by trained investigators. Health staff filled the questionnaires in the presence of the investigators. No incentives were given to participate in the surveys. Investigators were Lao doctors enrolled in the epidemiology module of a Masters degree course.

### Analysis

Data were double entered with Epidata (Odense, Denmark) and Stata, version 8 (Stata Corporation, College Station, TX). Chi-squared and Fisher's exact tests were used for categorical variables and Student's t test and analysis of variance (F test) for normally distributed continuous data. P < 0.05 was considered as significant.

### Ethical considerations

The survey was done with the agreement of the Ministry of Health, local and regional health authorities and the approval of the National Ethical Review Board of Laos. Families and health staff gave written informed consent to participate in the survey.

## Results

**Figure 2:**
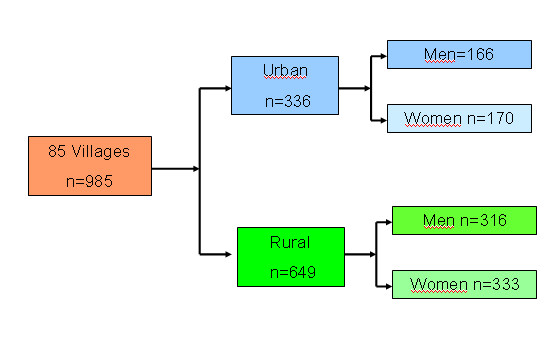
**Flow chart of general population**.

In March 2009, 985 adults were enrolled in 85 villages (Figure 2). None refused to participate. The mean age of participants was 39 years, the literacy rate was 72.6% (88.4% in urban areas), 65.8% were residents of rural areas and the majority (59%) were farmers. Only 49 (5%) had ever used the rectal route at home to treat their children and 93 (9.4%) to treat any family member, mostly enemas for constipation (using soap or local plants, 63.4%). There was no gender difference. Only 24.3% thought that rectal treatment could be a treatment for malaria and 96.8% would agree to use it for their children.

**Figure 3:**
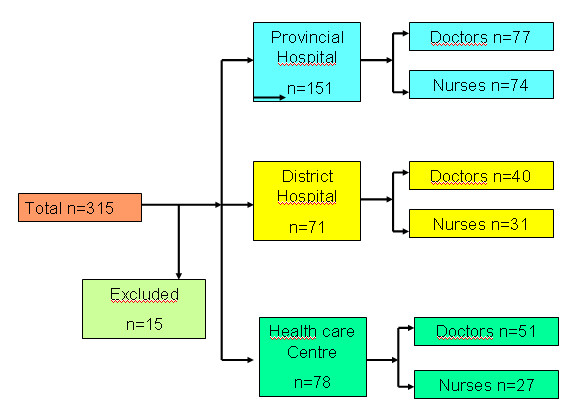
**Flow chart of health staff**.

Of 315 health staff interviewed, 15 were excluded subsequently since they were found to be part of the administrative rather than medical staff, leaving 300 staff (76.7% females with a mean 11.5 years of experience) for analysis (Figure 3). Overall, 90% had experience of using the rectal route, 25.3% knew about rectal artesunate or a rectal treatment for severe malaria when asked which treatment they specifically used for the rectal route. Among them, 48.7% had used paracetamol rectally and 17% had used rectal diazepam. More users were found in provincial hospitals than in the other sites. Figure 2 and 3 show the flow charts of health staff and population.

**Figure 4:**
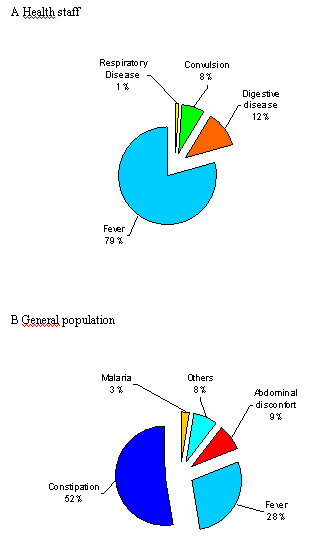
**Main indication for use of the rectal route among a) the general population and b) health staff**.

Health staff used the rectal route mainly to treat fever, while the population used it for constipation (Figure 4a and 4b). The population mostly used enemas and health staff mainly suppositories. Regarding the perceived efficacy of the rectal treatment route for malaria, 68% of the population surveyed thought it was less efficacious than other routes, 17% of equal efficacy, and 11% more efficacious. Out of the health staff questioned, 36% thought it was equally efficacious as other routes, 35% less efficacious and 28% more efficacious. The preferred treatment route for treating children with malaria among the general population was the oral route (42%) followed by parenteral therapy (39%: intravenous: 31%, intramuscular: 8%) and rectal treatment (4%). A few (15%) did not know how to answer the question. Table 1 summarizes common perceptions of problems associated with the rectal treatment route by the population.

**Table 1:** Opinion of the population of the rectal treatment route

Acceptability	Urban n = 336 (34.2%)	Rural n = 649 (65.8%)	*p*	Total n = 985
Difficult to do	138 (41.0)	215 (33.1)	0.01	353 (35.8)
Age to start rectal route (months)*	27.68 (23.6-31.7)	27.5 (24.7-30.6)	0.9	27.7 (25.3-30.0)
Age to stop (months)*	65.3 (51.3-79.2)	50.9 (43.4-58.4)	0.05	55.9 (49.0-62.8)
Will recommend to friends, yes	222 (66.0)	426 (65.6)	0.8	648 (65.7)
Will do rectal TT, after training	259 (77.0)	475 (73.1)	0.01	734 (74.2)

Perception	Urban n = 298 (37.9%)	Rural n = 489 (62.1%)	*p*	Total n = 787

Ineffective	216 (72.5)	318 (65)	0.03	534 (67.9)
Painful	102 (34.2)	122 (25)	0.005	224 (28.5)
Disliked by children	139 (46.6)	180 (36.8)	0.006	319 (40.5)
Difficult to perform	126 (42.3)	141 (28.8)	< 0.001	267 (33.69)
Adverse events	69 (23.1)	86 (17.6)	0.05	155 (19.7)
Leads to problems with defecation	82 (27.5)	105 (21.5)	0.053	187 (23.8)
Causes other diseases	57 (19.1)	59 (12.1)	0.007	116 (14.7)

* 95% confidence interval

Out of 300 health staff questioned, 149 working in peripheral hospitals and 151 in provincial hospitals, 153 (51%) reported having used the rectal route to administer treatment. The majority of these (97) worked in the provincial hospitals and had used the rectal route for drug administration 7.4[4.2-10.7] times on average in the previous two months compared to only 0.9[0.5-1.3] times in the 56 health care workers in peripheral hospitals, p = 0.02.

Health workers who had been working for less than 10 years were also more likely to use the rectal treatment route than those who had worked > 10 years, 56% vs 45% respectively. This difference was not statistically significant, however there was a significant difference in the number of uses in the previous 2 months between the 2 groups: 6.9[CI^95 ^3.4-10.4] compared to 2.3[1.5-3.1], p = 0.03. Drawbacks of the rectal route for drug administration were also described by many health-care workers, including inefficacy in case of diarrhoea (28%), poor hygiene (15.3%), pain (8%), discomfort in children (10%).

## Discussion

Treatment delays cost lives [8]. Access to effective treatment in remote areas needs to be improved. Rectal pre-referral treatment for malaria has been shown to be one strategy which may reduce mortality [4]. Adherence of health staff and population to referral advice is crucial and still a matter of debate [9,10].

Rectal pre-referral treatment for malaria has been adopted in Cambodia since 2000 and rectal artesunate for children has been implemented between 2005 and 2008 [11]. This study from neighbouring Laos has identified how local perceptions and traditional beliefs may restrict its uptake. Traditional use of enemas in some populations may be an advantage when use of rectal treatments is being promoted [12]. However, this did lead to a higher incidence of side-effects following rectal quinine in Africa since children had already some irritation of the rectum following traditional enemas [2].

Unlike other developing countries the survey found limited use of traditional rectal route, mainly enemas. Paracetamol and diazepam were given occasionally via the rectal route. Children, who are the target group of pre-referral treatment received rectal treatment infrequently. The population had limited confidence in the efficacy of the rectal route and made of number of a priori assumptions about lack of tolerability, which could hamper implementation.

Interestingly, while the rectal route was considered as a route for either young infants or elderly people, young adults with severe malaria were not mentioned as good candidates for treatment via this route, which warrants further investigation.

The main concern of the population regarding the use of rectal treatments was the potential to cause pain, discomfort or other side effects. In reality very few adverse events have been reported in studies of rectal artesunate [3]. The majority of the population also perceived the rectal route as less effective than alternative routes.

These findings highlight the need for education and improved communication if these treatments are to be implemented.

Concerns about lack of efficacy were shared by health care workers, although younger physicians were more likely to use the rectal route. Health staff from district hospitals had less experience of using the rectal route than those of provincial hospitals. In general they were not as highly trained as the latter and had less experience of using rectal diazepam (Lao doctors rarely use rectal diazepam and/or are not trained to use it to treat seizures, H. Barennes, personal observation, 2010). The survey findings also question the availability of rectal artesunate, which was supposed to be extended to all Lao provinces by 2005 after the change of policy [13]. ACT was not available at the village level during a previous survey, shortages were frequent and less than 30% of village health volunteers used them for confirmed malaria. Making ACT available at the village level remains a main challenge in many countries, e.g. less than 3% of children (ranging from 0.1% in The Gambia to 13% in Zambia) were receiving artemisinin-based combination therapy in 18 African countries in 2008 [14].

## Conclusion

Use of the traditional rectal route is infrequent in the general population in Laos. Overall it was perceived as a less effective treatment than other routes by the community and health care workers. Understanding perceptions related to the rectal route of the potential users is a key issue that might help to better implement of a pre-referral treatment and ensure good levels of uptake at the time of introduction. More information and training are needed to convince the population and health staff of the efficacy and advantages of the rectal route for malaria treatment.

## Competing interests

The authors declare that they have no competing interests.

## Authors' contributions

HB wrote the manuscript and is responsible for the overall coordination, design and analysis of the surveys in the Lao PDR. He is also guarantor. HB, SI, TF, YM, designed the survey. SI, TF, YM, collected the data and ensured data entries. HB performed the final analysis. EA participated to the design and writing of the manuscript. All authors contributed to the writing of the paper and read the final version.

## Acknowledgements

We thank the IFMT students (Dr S. Somphavong, V. Hansackda, B. Chaykaodaxue, Shi Jing, Lan Quing and all IFMT P10 class), IFMT staff, and assistants (A Hiarimanana, C Rajaonarivo, G Empis, K Paulin), and all the people who participated in collecting data during the surveys and all families participating in the surveys. We thank Dr Paul Newton for advice and Dr. P. Naphayvong and Dr P. Vongphrachanh for facilitating the relationship with Lao Authorities during the preparation of the survey and advice. We thank the Lao national and regional health authorities for their support and The Wellcome Trust and AUF/IFMT which funded the survey.
